# A Case of Long-Term Exposure to Valproic Acid Mimicking Tremor-Dominant Parkinson’s Disease

**DOI:** 10.5334/tohm.755

**Published:** 2023-05-15

**Authors:** Kazumasa Sekiguchi, Toshihiro Mashiko, Reiji Koide, Kensuke Kawai, Shigeru Fujimoto, Ryota Tanaka

**Affiliations:** 1 Division of Neurology, Department of Medicine, Jichi Medical University, Tochigi, Japan; 2 Department of Neurosurgery, Jichi Medical University, Tochigi, Japan

**Keywords:** Unilateral resting tremor, Parkinsonism, Valproic acid, Parkinson’s disease

## Abstract

**Background:**

Valproic acid is associated with increased risks of tremor and parkinsonism.

**Case Report:**

A 67-year-old man with a diagnosis of epilepsy who had been treated with valproic acid (VPA) for 32 years noticed right-dominant upper-limb resting tremor accompanied by mild rigidity and bradykinesia. He was initially diagnosed with tremor-dominant Parkinson’s disease (TDPD), but dopamine transporter single-photon emission computed tomography demonstrated no nigrostriatal degeneration. At 3 months after discontinuing VPA, his symptoms dramatically improved.

**Discussion:**

VPA-induced tremor usually consists of postural or kinetic tremor without asymmetry. Our case indicated that careful evaluation is needed, even in cases of asymmetrical resting tremor and mild parkinsonism resembling TDPD after long term exposure to VPA.

**Highlights:**

We report an atypical case of valproic acid-induced tremor and parkinsonism that mimics tremor-dominant Parkinson’s disease. Physicians should not exclude the possible relation to valproic acid in patients presenting unilateral resting tremor and parkinsonism even in the absence of long-term side effects.

## Background

Patients treated by antidopaminergic medications are at increased risk for drug-induced parkinsonism (DIP) [1,2]. Valproic acid (VPA), a classical type of anticonvulsant, has been widely used for not only epilepsy, but also bipolar disorder, post-traumatic stress disorder, and migraine. Patients being treated with VPA commonly experience tremor as a central nervous system side effect [3], and are at increased intermediate risk of DIP [2]. In addition, VPA has been reported to cause myoclonus, dystonia, dyskinesia, and other extrapyramidal symptoms [4]. The clinical features of VPA-induced tremor are usually postural or kinetic tremor of the upper or lower extremities without asymmetry [5], and a longer duration of VPA exposure may be a risk factor, but onset is typically several months to years after VPA initiation [3].

Here, we report a case of VPA-induced tremor and parkinsonism mimicking tremor-dominant Parkinson’s disease (TDPD) after 32 years VPA exposure.

## Case Report

**Figure 1:**
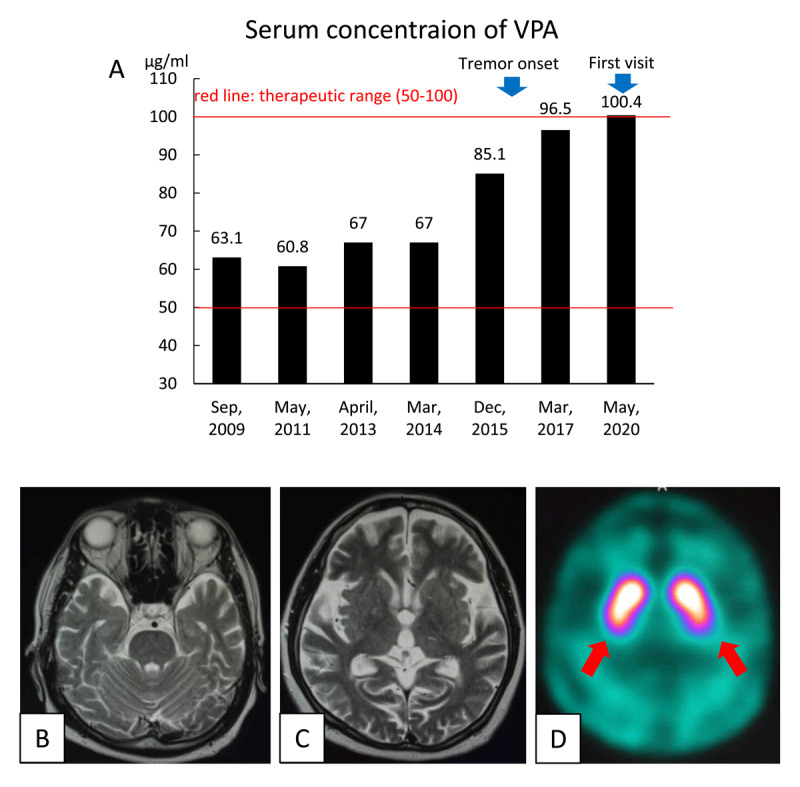
A) Bar graph shows changes in the serum concentration of valproic acid (VPA). The serum concentration of VPA was stable for long time, but rose preceding symptom onset (arrow on the lefts), and then gradually increased. The patient’s serum concentration was highest (100.4 μg/mL) at the time he visited our clinic (arrow on the rights). **B), C)** Brain magnetic resonance imaging showed only minor ischemic changes. **D)** [^123^I]b-CIT single-photon emission computed tomography showed no evidence of nigrostriatal degeneration.

A 67-year-old man who had been diagnosed with epilepsy and treated with VPA (1,200 mg/day) for 32 years noticed right-dominant upper-limb resting tremor that gradually developed and impaired his activities of daily living. Four years later, he presented to our clinic with right hand-dominant resting tremor, mild rigidity, and bradykinesia in both upper limbs, but showed normal posture and no apparent gait disturbance (Video 1). His score on the Movement Disorder Society (MDS)-Sponsored Revision of the Unified Parkinson’s Disease Rating Scale (MDS-UPDRS) part III was 18. A blood test showed normal thyroid gland function and no other abnormalities. Serum concentration of VPA was 60.8–67 µg/mL for an extended period, but rose prior to symptom onset and gradually increased in a time-dependent manner (Figure 1A). Electroencephalography showed no epileptic discharge and brain magnetic resonance imaging showed only minor ischemic change (Figure 1B, C). He also received the antipsychotic sulpiride (150 mg/day) for 32 yours for his depressive symptoms. As he showed right-dominant resting tremor, mild rigidity, and bradykinesia, the suspected diagnosis was TDPD. First, sulpiride was suspended for 1 month, but this did not improve his symptoms. As he requested resumption, the sulpiride was subsequently restarted. Because [^123^I]b-CIT single-photon emission computed tomography (SPECT) showed no reduction in dopamine transporter (DaT) activity (Figure 1D), he was suspected of having valproate-induced tremor and mild parkinsonism. Therefore, we discontinued VPA and replaced it with zonisamide (100 mg/day), the dose of which we increased to 200 mg/day 1 month later. At 1 month after stopping VPA, he showed no signs of improvement. However, his symptoms rapidly disappeared at 3 months later (Video 2) and his score on the MDS-UPDRS part III improved to 1 (facial expressions).

**Video 1:** **Initial presentation of this case.** The patient showed right hand-dominant resting tremor. When he raised both arms, his tremor was not exacerbated and was rather suppressed in the left arm. Finger tapping and pronation-supination movements of the hands showed broken regular rhythm or sight slowing. Posture and gait were almost normal, but right-dominant tremor continued when walking.

**Video 2:** **Three months after VPA discontinuation.** The patient’s-tremor completely disappeared and several maneuvers showed no apparent abnormality.

## Discussion

The present case showed several unique clinical features such as insidious development of right-dominant resting tremor, mild rigidity, and bradykinesia in both upper limbs that mimicked TDPD. The duration of symptom onset from first initiation of VA was very long.

The incidence of tremor in cases receiving VPA varies, but a recent meta-analysis reported an incidence of 14% [3]. VPA-induced tremor usually shows postural and kinetic tremor in the upper or lower limbs [5,6], but a more recent study found a higher occurrence of resting tremor in the upper limbs when compared with patients with essential tremor [7]. Parkinsonism involving bradykinesia, rigidity, and tremor has also been reported in about 1.4%–6.0% of patients taking VPA [8,9,10]. These symptoms usually occur months to years after the initiation of VPA treatment [3,8,9,10]. Although the clinical manifestation of DIP is usually characterized by symmetric symptoms and the absence of tremor, the presence of asymmetry and resting tremor make DIP difficult to distinguish from idiopathic Parkinson’s disease (IPD) [11]. TDPD is a distinct subtype from the akinetic-rigid form, and pronounced unilateral resting tremor usually affects the upper more than the lower limbs [12]. Tremor is provoked by stressful situations and suppressed during movement initiation [12]. These clinical characteristics resembled those of our case, and as a result, TDPD was the initial clinical diagnosis. Indeed, VPA-induced parkinsonism seems identical to IPD, and some cases treated with levodopa have shown marked improvement and dyskinesia [13]. Dopamine transporter (DAT)-SPECT is useful for discriminating DIP from IPD or underlying nigrostriatal degeneration [14]. Therefore, if the diagnosis is suspicious, DAT-SPECT should be actively evaluated. Age might have been risk factor for development of symptoms in the present case, as well as the higher dose of VPA (1,200 mg/day). A previous study reported that the plasma concentration of VPA varies at symptom onset, and 33 (89.2%) of 37 patients were within the recommended range for treating epilepsy (40–100 µg/mL) [15]. However, administrating higher daily doses of VPA, especially >1,000 mg/day, is an independent risk factor for VPA-induced tremor [3,16]. In the present case, the plasma concentration of VPA was evaluated regularly and remained within the recommended range, but incremental increases seemed to be associated with the onset and severity of symptoms (Figure 1A). Therefore, there may be an individual threshold regarding the onset of tremor or parkinsonism intoxication associated with VPA.

Although the mechanistic association between the use of valproic acid and parkinsonism remains unknown, these patients are at increased risk of developing Parkinson’s disease (PD) or may have coexistent early PD. However, our case showed no evidence of nigrostriatal degeneration on DAT-SPECT. VPA is commonly known to act by increasing levels of the inhibitory transmitter gamma-aminobutyric acid (GABA) via prevention of GABA degradation, suppression of GABA transamination action, and promotion of GABA synthesis [17]. Increased levels of GABA in the brain, such as in the substantia nigra and corpus striatum, as well as disturbances of the GABAergic pathways in the basal ganglia, may result in dopamine inhibition and subsequent changes in catecholamine [3]. Another possible pathological mechanism is that VPA may affect dopamine signaling by modulating the expression of associated genes and proteins [15]. This could explain the prolonged recovery period after discontinuation of VPA. Furthermore, the possibility of unmasking subclinical dopaminergic degeneration or enhanced neurodegeneration at the mitochondrial level has also been proposed [15].

VPA is still widely used in a variety of clinical settings. Therefore, clinicians should consider the possibility of VPA-induced tremor or parkinsonism, even in the absence of long-term side effects involving motor symptoms, marked asymmetry, and resting tremor resembling TDPD.

## References

[1] Bondon-Guitton E, Perez-Lloret S, Bagheri H, Brefel C, Rascol O, Montastruc JL. Drug-induced parkinsonism: a review of 17 years’ experience in a regional pharmacovigilance center in France. Mov Disord. 2011; 26(12): 2226–31. DOI: 10.1002/mds.23828 21674626

[2] López-Sendón JL, Mena MA, de Yébenes JG. Drug-induced parkinsonism in the elderly: incidence, management and prevention. Drugs Aging. 2012; 29(2): 105–18. DOI: 10.2165/11598540-000000000-00000 22250585

[3] Zhang CQ, He BM, Hu ML, Sun HB. Risk of Valproic Acid-Related Tremor: A Systematic Review and Meta-Analysis. Front Neurol. 2020; 11: 576579. DOI: 10.3389/fneur.2020.576579 33384651 PMC7769765

[4] Rissardo JP, Fornari Caprara AL, Durante Í. Valproate-associated Movement Disorder: A Literature Review. Prague Medical Report. 2021; 122: 140–180. DOI: 10.14712/23362936.2021.14 34606429

[5] Alonso-Juarez M, Torres-Russotto D, Crespo-Morfin P, Baizabal-Carvallo JF. The clinical features and functional impact of valproate-induced tremor. Parkinsonism Relat Disord. 2017; 44: 147–50. DOI: 10.1016/j.parkreldis.2017.09.011 28941829

[6] Alonso-Juarez M, Baizabal-Carvallo JF. Distinguishing features between valproate-induced tremor and essential tremor. Acta Neurol Scand. 2018; 138(2): 177–81. DOI: 10.1111/ane.12953 29749618

[7] Paparella G, Angelini L, De Biase A, Cannavacciuolo A, Colella D, Di Bonaventura C, et al. Clinical and Kinematic Features of Valproate-Induced Tremor and Differences with Essential Tremor. Cerebellum. 2021; 20(3): 374–83. DOI: 10.1007/s12311-020-01216-5 33200286 PMC8213593

[8] Easterford K, Clough P, Kellett M, Fallon K, Duncan S. Reversible parkinsonism with normal beta-CIT-SPECT in patients exposed to sodium valproate. Neurology. 2004; 62(8): 1435–7. DOI: 10.1212/01.WNL.0000121228.32913.00 15111693

[9] Ristić AJ, Vojvodić N, Janković S, Sindelić A, Sokić D. The frequency of reversible parkinsonism and cognitive decline associated with valproate treatment: a study of 364 patients with different types of epilepsy. Epilepsia. 2006; 47(12): 2183–5. DOI: 10.1111/j.1528-1167.2006.00711.x 17201721

[10] Jamora D, Lim SH, Pan A, Tan L, Tan EK. Valproate-induced Parkinsonism in epilepsy patients. Mov Disord. 2007; 22(1): 130–3. DOI: 10.1002/mds.21188 17115396

[11] Tinazzi M, Ottaviani S, Isaias IU, Pasquin I, Steinmayr M, Vampini C, et al. [123I]FP-CIT SPET imaging in drug-induced Parkinsonism. Mov Disord. 2008; 23(13): 1825–9. DOI: 10.1002/mds.22098 18759353

[12] Chen W, Hopfner F, Becktepe JS, Deuschl G. Rest tremor revisited: Parkinson’s disease and other disorders. Transl Neurodegener. 2017; 6: 16. DOI: 10.1186/s40035-017-0086-4 28638597 PMC5472969

[13] Silver M, Factor SA. Valproic acid-induced parkinsonism: levodopa responsiveness with dyskinesia. Parkinsonism Relat Disord. 2013; 19(8): 758–60. DOI: 10.1016/j.parkreldis.2013.03.016 23632325

[14] Eerola J, Tienari PJ, Kaakkola S, Nikkinen P, Launes J. How useful is [123I]beta-CIT SPECT in clinical practice? J Neurol Neurosurg Psychiatry. 2005 Sep; 76(9): 1211–6. DOI: 10.1136/jnnp.2004.045237 16107353 PMC1739796

[15] Brugger F, Bhatia KP, Besag FM. Valproate-Associated Parkinsonism: A Critical Review of the Literature. CNS Drugs. 2016; 30(6): 527–40. DOI: 10.1007/s40263-016-0341-8 27255404

[16] Lan L, Zhao X, Jian S, Li C, Wang M, Zhou Q, Huang S, Zhu S, Kang H, Kirsch HE. Investigation of the risk of valproic acid-induced tremor: clinical, neuroimaging, and genetic factors. Psychopharmacology (Berl). 2022; 239: 173–184. DOI: 10.1007/s00213-021-06004-5 34718848

[17] Silva MF, Aires CC, Luis PB, Ruiter JP, L IJ, Duran M, Wanders RJ, et al. Valproic acid metabolism and its effects on mitochondrial fatty acid oxidation: a review. J Inherit Metab Dis. 2008; 31(2): 205–16. DOI: 10.1007/s10545-008-0841-x 18392741

